# Alcohol use in adult patients with autism spectrum disorder (ASD). Case report

**DOI:** 10.1192/j.eurpsy.2023.1606

**Published:** 2023-07-19

**Authors:** P. Del Sol Calderon, A. Izquierdo de la Puente, A. Alvarez Astorga, M. García Moreno, R. Fernandez Fernandez

**Affiliations:** 1 Psychiatry, Hospital Universitario Puerta de Hierro; 2 Psychiatry, Hospital Puerta de Hierro; 3 Psychiatry, Hospital Infanta Leonor; 4 Psychiatry, Hospital Infanta Cristina, Madrid, Spain

## Abstract

**Introduction:**

Patients with autism spectrum disorder are characterized by high anxiety when facing social situations and dealing with interpersonal relationships on a daily basis. Although initially because of their rigid personality with the norm, and their tendency to social distancing, we do not have in mind this pathology as the most likely to develop a substance use disorder. However, it is observed in the literature a remarkable percentage of patients who resort to consumption, mainly alcohol, as an anxiolytic to be able to interact in society.

**Objectives:**

To show the case of a 19-year-old adult with a diagnosis of ASD who resorts to alcohol consumption in her daily life as a strategy to manage anxiety in social situations.

**Methods:**

Case report and literatura review

**Results:**

This is a 19-year-old woman with a recent diagnosis of ASD. She is studying biotechnology and lives with her parents and 3 siblings. The patient reports difficulty in social relationships since early childhood, with experiences of school bullying. She expresses desire to relate with others, although she does it in an inadequate way, with difficulty in detecting nonverbal language, irony and anger when she does not understand a joke. The patient confesses that since she was 16 years old she has consumed alcohol to mitigate the anxiety caused by facing a group of people. She says that she feels that it relaxes her and facilitates interaction, making it more fluid and less tense. However, she recognizes that initially she used to drink 1 or 2 beers, but now she needs to drink up to 2 glasses of gin, recognizing this as something problematic.

**Conclusions:**

The literature shows how patients with ASD can also present substance use disorder. It has been shown that about 10% of these patients have an abusive use of alcohol. Other samples show wider ranges (7-71%) of prevalence of alcohol consumption in patients with autism. In relation to cannabis, it is seen that around 3% of these patients consume it. These patients seek its anxiolytic effect and to reduce mental health symptoms. In addition, the purchase of alcohol does not involve high social interaction to obtain it, since it is a substance that can be purchased legally. It is important to explore alcohol consumption in consultation with patients with ASD to help them develop more functional anxiety management strategies.

**Reference:**

Prevalence of psychiatric disorders in adults with autism spectrum disorder: A systematic review and meta-analysis. Lugo-Marín.J et al. 2019. Research in Autism Spectrum Disorders Volume 59, March 2019, Pages 22-33

**Disclosure of Interest:**

None Declared

